# Quality assessment of the registration of vulvar and vaginal premalignant lesions at the Cancer Registry of Norway

**DOI:** 10.3109/0284186X.2011.624545

**Published:** 2011-11-02

**Authors:** Espen Enerly, Freddie Bray, Christine Mellem, Bo Terning Hansen, Grete KjøLberg, Tove Dahl, Tom Børge Johannesen, Mari Nygård

**Affiliations:** 1 Cancer Registry of Norway, Oslo University Hospital, Post Box 5313 Majorstuen, 0304 Oslo, Norway; 2 Section of Cancer Information, International Agency for Research on Cancer, Lyon, France

## Abstract

**Background:**

A crucial factor concerning the utility of Cancer Registries is the data quality with respect to comparability, completeness, validity and timeliness. However, the data quality of the registration of premalignant lesions has rarely been addressed. High grade vulvar intraepithelial neoplasia (VIN) and vaginal intraepithelial neoplasia (VaIN) are premalignant lesions which may develop into cancer, and are often associated with infection with the human papillomarvirus (HPV). The aim was to evaluate the quality of registration of VIN and VaIN at the Cancer Registry of Norway (CRN).

**Material and methods:**

We re-collected all notifications with high grade VIN and VaIN diagnoses during 2002 to 2007 from pathology laboratories, and compared these to the data in the CRN database so as to quantitatively measure the completeness, validity and timeliness of the data.

**Results:**

Over the period 2002 to 2007 we estimated the completeness of the 1556 VIN and 297 VaIN notifications to be 95.0% and 92.9%, respectively. The original and reabstracted topography codes showed major discrepancies for 12 of 642 (1.9%) VIN and 7 of 128 (5.5%) VaIN notifications. The original and reabstracted morphology codes for VIN and VaIN were identical for 724 out of 814 notifications. Sixteen notifications had a major discrepancy. For the period 2002 to 2007 the median time elapsed between date of diagnosis and date of registration were 436 and 441 days for VTN and VaIN cases, respectively.

**Discussion:**

Based on the present analysis of the comparability, completeness, validity and timeliness of premalignant lesions of vulva and vagina, we conclude that the Cancer Registry of Norway is able to monitor such premalignant lesions satisfactorily.

In recent decades the role of Cancer Registries has expanded beyond the generation of descriptive statistics and may also include planning, monitoring and evaluation of cancer control activities, including cancer screening and vaccination programmes, and the follow-up of the quality of care for cancer patients [1]. A crucial factor concerning the utility of the Registry in such activities is the quality of the data with respect to comparability, completeness, validity and timeliness [2,3].

In Norway, it has been compulsory to send notifications of malignant and premalignant lesions to the Cancer Registry of Norway (CRN) since 1952 [4]. Data quality evaluation has been applied to several specific cancers, including cancer of the ovary [5], prostate [6], central nervous system [7] and pharynx [8]. A recent comprehensive evaluation of the data quality of solid and non-solid tumours concluded that data from CRN yields comparable data that can be considered reasonably accurate, timely and close to complete [9]. However, the use of such concepts in the evaluation of the data quality of registration of premalignant lesions has rarely been applied.

Norway has recently introduced a nationwide vaccination programme against HPV, the main causative agent for cervical cancer. In several clinical trials, vaccination has reduced the incidence of cervical intraepithelial neoplasia [10,11], a precursor to cervical cancer. HPV also causes premalignant lesions in the vulva and vagina [12,13]. High grade vulvar intraepithelial neoplasia (VIN) and vaginal intraepithelial neoplasia (VaIN) are premalignant lesions which may develop into cancer or spontaneously regress [14,15]. In Norway, the incidence rate of vulvar intraepithelial neoplasia increased three-fold from 1973-1977 to 1988-1992 [16].

The aim of the present study was to assess the data quality for the registration of premalignant lesions of high grade VIN and VaIN at the CRN. Monitoring the incidences of these premalignant lesions is important to appropriately evaluate the impact of HPV vaccination in the population. We re-collected all notifications with high grade VIN and VaIN diagnoses during 2002 to 2007 from pathology laboratories, and compared these to the data in the CRN database so as to quantitatively measure the validity and completeness of the data. We also present the comparability and timeliness of VIN and VaIN registrations at the Registry.

## Material and methods

### Source of information

The Cancer Registry of Norway (CRN) collects, codes, and stores data on patients with malignant and certain premalignant diagnoses by combining information from different sources; pathology notifications, clinical notifications, death certificates, hospital discharge diagnoses and radiation therapy data. Coding and classification of neoplasms at the CRN have been described elsewhere [9]. The major sources of information for premalignant lesions are pathology notifications from hospitals and private pathology laboratories that send copies of histology reports routinely to CRN. These notifications also have topography and morphology codes from the Norwegian version of the Systematised Nomenclature of Medicine (SNOMED). At CRN the notifications are registered by a trained medical coder who assigns topography and morphology codes according to the rules and classification schemes used at CRN which are mainly based on the second edition of the International Classification of Diseases for Oncology (ICD-O-2). Notifications are scanned and stored in the database and accumulated as one record for each patient. This is effectuated by using the 11-digit personal identification number (PIN) issued to every newborn Norwegian citizen and to people residing in Norway.

### Histological features of high grade VIN and VaIN

Vulvar intraepithelial neoplasia (VIN) is a disease which shows histological features of disordered maturation and nuclear abnormalities, such as loss of polarity, pleomorphism, coarse chromatin, irregular nuclear membranes and mitotic figures [17]. Warty, basaloid and mixed warty-basaloid are histological subtypes of HPV-related VIN, and are referred to as high grade VIN (also known as usual VIN (classic) VIN2-3 or undifferentiated VIN) [18]. In contrast, the differentiated VIN type is not associated with HPV. The distinction between high grade VIN and differentiated VIN is not registered at the CRN. Women with HPV-related vulvar disease have an increased risk of concurrent or subsequent cervical intraepithelial and vaginal intraepithelial neoplasia [15]. The histopathological features of VaIN are similar to those of VIN and include hyperkeratosis, nuclear enlargement and pleomorphism. We use VIN as a synonym for high grade VIN and VIN 2/3, as recommended by Sideri et al. [19] and we use VaIN as a synonym for high grade VaIN and -VaIN 2/3.

### Quality measurements

#### Comparability

The comparability of registry data can be defined as the extent to which coding and classification procedures, and the definitions for recording of specific data items adhere to agreed international standards [2]. The topics covered here include the definition of incidence of premalignant lesions, incidence date of the lesion and definition of multiple lesions either at the same site (topography) or at other anatomically close locations.

#### Completeness

The completeness of cancer registry data reflects the extent to which all diagnosed incident cancer cases occurring in the population are included in the registry database [3]. To obtain a quantitative measurement of the degree of completeness, we performed independent case ascertainment by re-collecting notifications from the pathology laboratories. We contacted all of the 23 Norwegian pathology laboratories and requested them to re-identify and send to CRN all pathology notifications with histologically verified VIN and VaIN for the period 2002 to 2007. A predefined list of the Norwegian SNOMED codes for VIN and VaIN (Table I) with corresponding WHO descriptions of VIN and VaIN, was provided in the request. The period 2002 to 2007 was chosen to: 1) allow the hospital laboratories to use electronic systems to identify premalignant cases of the selected topographies and morphologies; and 2) to ensure that the quality of data for estimating VIN and VaIN incidences in the pre-vaccination era would be satisfactory.

**Table I:** From the list of morphology codes for vagina and vulvar sites with terminology descriptions provided for the 23 laboratories during re-collection of notifications based on Norwegian SNOMED laboratory coding lists.

Codes	Terminology/Description
80102	Carcinoma in situ, NOS^a^ (Intraepithelial carcinoma, NOS)
80502	Papillary carcinoma in situ
80522	Papillary squamous cell carcinoma, non-invasive
80522	Papillary squamous cell papilloma with atypia
80522	Carcinoma in situ in squamous cell papilloma
80702	Squamous cell carcinoma in situ, NOS
80702	Severe dysplasia
80702	Squamous intraepithelial neoplasia
80762	Squamous cell carcinoma in situ with questionable stromal invasion (not microinvasion)
80812	Bowen disease
81402	Adenocarcinoma in situ NOS. Severe dysplasia in glandular epithelium, NOS
74000	Dysplasia, NOS
76086	Mild/moderate dysplasia in glandular epithelium
Others	Any other code used locally to represent moderate or severe dysplasia, carcinoma in situ or intraepithelial neoplasia, VIN or VaIN.

The ICD-O/WHO code 80772 is not used in the Norwegian laboratory lists.

a Not otherwise specified.

#### Validity (accuracy)

Validity is the proportion of cases in a dataset with a given characteristic which truly have the attribute [2]. We identified all notifications routinely sent to the registry as well as those recollected following our request. From the latter we further selected a random subset of notifications for the medical coders to reabstract. During reabstrac-tion, the medical coders had on-screen information of the patient record available, but did not consult previously registered codes for the notification at hand. This method was chosen to mimic the setting in which the codes were originally recorded. We then compared the data registered at CRN (the original codes) with the data reabstracted from notifications re-collected from the pathology laboratories (the reabstracted codes). The accuracy analysis of morphology coding was performed on 814 (69%) randomly chosen notifications.

A total of 44 VaIN notifications had an original code of cervical topography and a reabstracted code of vaginal topography. This disparity was caused by a change of coding practice in 2009, before reabstraction, and therefore was not due to an error in coding. These notifications were excluded from the accuracy analysis of topography coding, leaving 128 VaIN notifications for analyses. The accuracy analysis of vulvar topography coding was performed on 642 notifications.

All discrepancies between original and reabstracted codes for each record were classified according to severity. We defined a major topographic discrepancy as any change in coding of sites. For a vulvar lesion a minor discrepancy was defined as variation within vulvar topographies, such as labium majus, labium minus, unspecified labium majus/ minus and unspecified vulva.

We further defined a major morphologic discrepancy as any coding change between low grade lesions, high grade lesions and carcinoma. Minor morphologic discrepancies were defined as use of different codes to describe high grade VIN and VaIN. These distinctions are modified from Havener [20] with the intention of emphasising discrepancies which would affect incidence trend estimates.

#### Timeliness

Timeliness is considered here as the time from diagnosis to registration at CRN for each notification. The date of diagnosis is the date at which the sample was taken. We estimated timeliness based on all VIN and VaIN notifications over the registration period 2002 to 2007. Data was extracted from the CRN database in November 2009.

## Results

### Comparability

A patient was defined as an “incident premalignant case” upon registration of a new diagnosis of high grade VIN (or VaIN) with no history of histologically confirmed high grade lesion or cervical cancer at the same anatomical site in the past two calendar years. Women with a cancer diagnosis within four months subsequent to the high grade lesion were excluded, as these were considered “missed” cases of invasive cancer.

The CRN rule for the registration of incidence date of malignancies is to register the earliest date reported for a confirmed malignant diagnosis and the incident date for premalignant VIN and VaIN cases are similarly defined as the earliest date on notifications describing an incident premalignant case.

The coding of multiple primary tumours mainly follows the recommendation given by the European Network of Cancer Registries (ENCR), using groups of topography codes considered as single sites (C51-vulva and C52-vagina) and that the recognition of two or more primary cancers is not time dependant. The CRN has in the period 1974 to 2007 taken into account the premalignant/malignant history of the woman when assigning topography to premalignant lesions of vulva and vagina. The registration of a cancer or premalignant lesion in an adjacent site prior to the new diagnosis usually resulted in the new diagnosis being assigned to the adjacent site. However, no clear rules were established at that time, leaving it up to the medical coder at CRN to interpret and assign the topography code.

### Completeness

For the period 2002 to 2007 we performed independent case ascertainment. The records created from routinely registered pathology notifications in the CRN database were compared to records created from the notifications re-collected from the pathology laboratories. A total of 1853 notifications were analysed and showed that 78 of 1556 (5.0%) VIN and 21 of 297 (7.1%) VaIN notifications were missing in the Registry, giving estimates of 95.0% and 92.9% completeness for VIN and VaIN notifications, respectively. There was some variations in completeness over the observed period, both for VIN (2002: 96.3%, 2003: 96.2%, 2004: 96.1%, 2005: 93.4%, 2006: 97.9%, 2007: 89.8%) and for VaIN (2002: 94.1%, 2003: 93.6%, 2004: 92.5%, 2005: 92.9%, 2006: 92.9%, 2007: 91.7%). The completeness was lowest in 2007 at both sites. We also observed that the missing notifications were quite evenly distributed among the 23 pathology laboratories, except for one laboratory which submitted only 19 of 30 (63%) of their notifications (data not shown).

**Figure 1:**
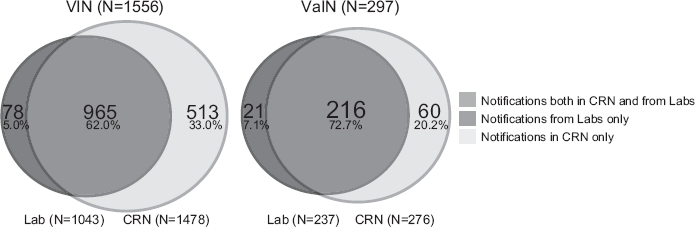
The completeness of the pathology notifications of VIN (Vulvar Intraepithelial Neoplasia) and VaIN (Vaginal Intraepithelial Neoplasia) registered at the Cancer Registry of Norway during 2002 to 2007. “Notifications in CRN only” refers to those notifications that were not sent from pathology laboratories during the independent case ascertainment.

We noticed that 573 more notifications (including both VIN and VaIN) were found in the registry than were received from the laboratories during re-collection (Figure 1). Of the 573 notifications, 184 were coded to the anatomical site skin by the pathology laboratories before being coded to VIN or VaIN by the medical coders at CRN (Supplementary Table I available at http://www.informahealthcare.com/doi/abs/10.3109/0284186X.2011.624545). In addition, 114 of these 573 notifications had other laboratory SNOMED codes than requested in the list of morphology codes sent to the laboratories (Table I). For the period 2002 to 2007 the average number of notifications per female was 1.9 for VIN, and 2.1 for VaIN. Twenty-five women had at least one VIN and one VaIN diagnosed.

### Validity (accuracy)

The original and reabstracted topography codes for VIN were identical for 593 of 642 notifications (92.4%). Minor and major discrepancies between the original coding and reabstracted coding was evident for 37 (5.8%) and 12 notifications (1.9%), respectively (Table II). No particular pattern of misclassification of vulvar topography codes was evident among the 37 notifications with minor discrepancy between the original and reabstracted topography (Supplementary Table II available at http://www.informahealthcare.com/doi/abs/10.3109/0284186X.2011.624545). The original and reabstracted topography codes for VaIN were identical for 121 of 128 notifications (94.5%) while seven notifications (5.5%) had a major discrepancy between the original coding and reabstracted coding (Table II).

**Table II:** Accuracy of topography and morphology codes assigned to notifications at Cancer Registry of Norway (CRN) and reabstracted notifications.

		Original versus reabstracted coding					
		
	Total	Identical		Major discrepancy		Minor discrepancy	
				
	N^a^	N	%	N	%	N	%
Topography^b^							
Vulva	642	593	92.4	12	1.9	37	5.8
Vagina	128	121	94.5	7	5.5	0	0
Total	770	714	92.7	19	2.5	37	4.8
Morphology							
Vulva	642	582	90.7	14	2.2	46	7.2
Vagina	172	142	82.6	2	1.2	28	16.3
Total	814	724	88.9	16	2.0	74	9.1

a number of notifications.

b Discrepancies are assigned to vulva or vagina according to the topographic code on the reabstracted notification.

The original and reabstracted morphology codes for VIN and VaIN were identical for 724 of 814 notifications. A minor discrepancy between the original coding and reabstracted coding was evident for 74 notifications (9.1%). A major discrepancy was found for 16 notifications (2.0%), of which 11 (1.4%) were coded as carcinomas and five (0.6%) as low grade lesions (Supplementary Table III available at http://www.informahealthcare.com/doi/abs/10.3109/0284186X.2011.624545).

We observed that 19 premalignant cases were registered with a topography code according to ICD-7 only and not according to ICD-O-2 in addition. These were patients with previous diagnoses coded according to ICD-7. We did not identify any missing morphology data at CRN on the notifications examined.

### Timeliness

For the 1478 VIN and 276 VaIN notifications registered at CRN the median time between date of diagnosis and date of registration was 436 (range: 29-2345) and 441 (59-1119) days for VIN and VaIN cases, respectively. In the period 2002 to 2006, the time interval decreased from 739 (58-2345) to 267 (29-921) days. In 2007 the median time interval increased to 445 (59-1119) days.

## Discussion

To our knowledge, this is the first study to report estimates of completeness for the registration of VIN and VaIN at a Cancer Registry. We can therefore only compare our estimates to those reported for cancers. Larsen et al. [9] estimated the average completeness of invasive vulvar and vaginal cancer incidence registration at CRN to be 99.8% [9]. Our estimates of completeness for the period 2002 to 2007 for VIN and VaIN were 95.0% and 92.9%, respectively, with some variation between years. One reason for the somewhat lower completeness of premalignant lesions than of cancer may be that some cancers are obtained from death certificates. Cancer mentioned on the death certificate allows CRN to send a reminder to the hospitals that have not yet reported a new cancer case [9]. It should also be noted that there is some uncertainty in the completeness estimates reported here since the pathology laboratories during re-collection of notifications did not manage to identify at least 573 notifications that were previously registered as VIN or VaIN in the CRN database. It is thus possible that they also may have failed to submit notifications not already registered by the CRN. The highest percentage of notifications missing in the CRN database occurred in 2007. This may indicate that a small proportion of the 2007 notifications were not yet registered in the database at CRN at the time of data extraction. This is in line with the observation that timeliness for VIN and VaIN was 267 days in 2006, compared to 445 days in 2007. The registration of a subset of VIN and VaIN notifications from 2007 was put on hold due to internal priorities at CRN and may explain some of the increase. The time interval from diagnosis to the notifications is sent to CRN by the pathology laboratories could be another source of variation in timeliness.

A total of 184 lesions that were assigned skin SNOMED topography codes by the pathology laboratories were coded to vulva topography by the medical coders at the CRN. This highlights the importance of a national cancer registry that can harmonise the coding practices of local pathology laboratories. Moreover, it illustrates how discrepancies in coding practices may influence completeness estimates in quality assessments of disease registration.

A VIN or VaIN lesion in a patient with a prior premalignant or malignant lesion in a nearby site has generally been assigned to the topography of the primary registration by the CRN medical coders. This has led to fewer registrations of VaIN in particular. A reason is that far more lesions occur in the cervix [21] and it is therefore more likely that a VaIN lesion has been assigned to the cervix than a cervical lesion has been assigned to the vagina. We have changed the coding practice at CRN so that VaIN and VIN cases are assigned topography codes according to the site of the lesion, regardless of disease history at other anatomical locations. The change took effect in 2009, and we will also recode retrospectively for the years 2002 to 2008. We believe that this change of practice will give us more reliable data to monitor the results of HPV vaccinations on the incidence of HPV-related diseases of the vulva and vagina. This change of coding practice affected 44 notifications with lesions originally assigned cervical topography code, but during the reabstraction assigned vaginal topography code. These were not included in the estimation of the accuracy of topography codes assigned to notifications at CRN and reabstracted notifications.

We have modified the published definitions of the severity of misclassification of cancers to evaluate the accuracy in registration of premalignant lesions. In brief, a major discrepancy as opposed to a minor discrepancy affects the incidence rates of VIN and VaIN. We could not compare the accuracy of the CRN coding of VIN and VaIN to other cancer registries since no such data, to our knowledge, has been published.

We found that 9.1% of the notifications had a minor discrepancy between the original and reabstracted morphology codes. Minor discrepancies occurred even though rules for giving priority to some codes over others exist for diagnoses with more than one appropriate morphology code. The majority of minor discrepancies occurred on notifications describing VIN 3/VaIN 3, carcinoma in situ and severe atypia/dysplasia in squamous epithelial cells for which there are separate morphology codes. The number of minor discrepancies should be reduced to a minimum by changing the coding practice. This entails using fewer morphology codes.

The fact that 1.5% VIN and 2.2% VaIN of all notifications had a major discrepancy between the original and reabstracted morphology and topography codes, indicate that major misclassification is not a significant problem in the registration of high grade VINandValN.

The selection of morphology codes used in this evaluation of data quality was chosen to assess the feasibility of the CRN to effectively monitor how HPV vaccination programmes will impact on the incidence of HPV-related premalignant diseases of the vulva and vagina. The present analysis indicates that the comparability, completeness, validity and timeliness of high grade VIN and VaIN registration is satisfactory, and therefore that the Cancer Registry of Norway is able to monitor high grade vulvar intraepithelial and vaginal intraepithelial neoplasias with adequate precision.

## Supplementary Material

Supplementary Tables I-III
