# Supplementary material for: Quality assessment of the registration of vulvar and vaginal premalignant lesions at the Cancer Registry of Norway
Source: Acta Oncol. 2011 Nov 2;51(1):45–50. doi: 10.3109/0284186X.2011.624545 (PMC3251004; doi:10.3109/0284186X.2011.624545)
Supplement: Supplementary Tables I-III [file 624545_suppl.pdf]

Supplementary material for Espen Enerly et al., Quality assessment of the registration of vulvar and vaginal premalignant lesions at the Cancer Registry of Norway, Acta Oncologica, 2012;51:45–50.

Supplementary Table I. Distribution of topography codes used by Pathology laboratories and Cancer Registry of Norway (CRN) for notifications not received from laboratories during reabstraction.

	From CRN[b]	
From pathology laboratories[a]	Vulva	Vagina
Vulva	277	4
Vagina	12	39
Skin	183	1
Cervix	8	9
Other[c]	14	4
NA[d]	20	2
Total	514	59

[a]SNOMED topography codes are replaced by corresponding topography descriptions.
[b]ICD-O-2 topography codes are replaced by corresponding topography descriptions.
[c]Anus, uterus, perineum, endometrium and urethra.
[d]Not available, No SNOMED code on the notification.

Supplementary Table II. Comparison of topography coding between original and reabstracted Cancer Registry of Norway (CRN) codes.

		Reabstracted codes					
		**Vulva Labium majus**	**Vulva Labium minus**	**Vulva NOS labium majus/minus**	**Vulva NOS**	**Vagina**	**Cervix**
**Original codes**	**Vulva Labium majus**	9			8		
	**Vulva Labium minus**		22		12		
	**Vulva NOS[a] labium majus/minus**			1	1		
	**Vulva NOS**	4	12		561	5	2
	**Vagina**				2	121	
	**Cervix**				3	44	
	**Others[b]**				5	2	

Discrepancies between original code and reabstracted code are highlighted in light gray (minor discrepancy) and dark gray (major discrepancy). ICD-O-2 topography codes are replaced by corresponding topography descriptions.
[a]Not otherwise specified.
[b]Skin, anus, urethra and corpus uteri.

Supplementary Table III. Comparison of morphology coding between original and reabstracted Cancer Registry of Norway (CRN) codes.

	Reabstracted codes									
Original codes	8077/29	8070/29	7063/19	7062/19	7069/19	7231/29	7060/19	8081/29	Carcinoma	Mild atypia in SEC
8077/29	478	5	2		2	10			1	
8070/29	8	92		1	2					
7063/19	10	2	81	1	3					
7062/19	3	1		32	3	4				
7069/19	3	3		1	30	1	1		1	
7231/29	2									
7060/19					2					
7032/19										1
7033/19			1							
8140/29					1					
8001/19					1					
8081/29		1						11		
Carcinoma[a]	7		1		1					
Mild atypia in SEC[b]				1	2	1				

Discrepancies between original code and reabstracted code are highlighted in light gray (minor discrepancy) and dark gray (major discrepancy). Code descriptions of codes used at CRN: 807729 (VIN3 or VaIN3), 807029 (Squamous cell carcinoma *in situ*), 706319 (Severe atypia/dysplasia in squamous epithelial cells), 706219 (Moderate atypia/dysplasia in squamous epithelial cells), 706919 (Atypia/dysplasia in squamous epithelial cells, NOS, precancerous), 723129 (High grade squamous intraepithelial lesion HSIL, VIN 2/3 or VaIN2/3), 706019 (Irregular squamous epithelium, uncertain atypia), 703219 (Moderate atypia/dysplasia in glandular epithelium), 703319 (Severe atypia/dysplasia in glandular epithelial cells), 814029 (Adenocarcinoma *in situ*), 800119 (Suspect malignant cells), 808129 (Bowen disease).

[a]Basaloid carcinoma, squamous cell carcinoma, microinvasive squamous cell carcinoma.

[b]Squamous epithelial cells.
